# The corrosion behavior of low carbon steel (AISI 1010) influenced by grain size through microstructural mechanical

**DOI:** 10.1038/s41598-023-47744-y

**Published:** 2024-03-01

**Authors:** Sayer Obaid Alharbi, Shakeel Ahmad, Taza Gul, Ishtiaq Ali, Abdul Bariq

**Affiliations:** 1 Mathematics Department, College of Science Al-Zulfi, Majmaah University, 11952 Majmaah, Saudi Arabia; 2 Department of Mechanical Engineering, University of Engineering and Technology, Peshawar, Pakistan; 3 Department of Mathematics, City University of Science and Information Technology, Peshawar, 25000 Pakistan; 4 Department of Mathematics and Statistics College of Science, King Faisal University, P. O. Box 400, 31982 Al-Ahsa, Saudi Arabia; 5 Department of Mathematics, Education Faculty, Laghman University, Mehtarlam, 2701 Laghman Afghanistan

**Keywords:** Energy science and technology, Engineering, Materials science

## Abstract

Low-carbon steel (AISI 1010) is the predominant material used in industrial food processing equipment. Such equipment is vulnerable to the corrosive environment produced by various production stages. Different processes, such as sulphonation and carbonation, are used in the processing of sugar in the sugar industry, creating a corrosive atmosphere. The corrosion behavior of low carbon steel (AISI 1010) is strongly influenced by grain size variations, which in turn affect the microstructural mechanical properties of the material. The mechanical behavior and performance of metallic materials, including their corrosion resistance, is determined by grain size which is an important parameter for this phenomena. The impact of low-carbon steel (AISI 1010) microstructure on corrosion behavior is discussed in this work. Heat treatment produces two different types of microstructure from the same material, which are then analyzed. Scanning Electron Microscopy (SEM) and Energy Dispersive Spectroscopy (EDS) have both been used to study characteristics including morphology and content. By supplying an appropriate corrosive medium, the corrosion performance of several microstructures of low-carbon steel (AISI 1010) was assessed, and corrosion rates were calculated using weight-loss and electrochemical techniques. Results show that the creation of a protective coating with a higher charge transfer resistance is caused by the adsorption process. The variety in phases and grain sizes may contribute to the corrosion stability of different microstructures, and as a result, the corrosion rate lowers as average grain sizes are reduced. Employing the galvanic effect, pearlite increases the rate of ferrite corrosion. The study's findings support the notion that quenching low-carbon steel (AISI 1010) results in a finer grain structure and greater corrosion resistance.

## Introduction

Smaller grain sizes are usually associated with improved mechanical properties, such as higher strength and hardness. The material's overall strength is enhanced because of the more grain boundaries, which serve as barriers to the migration of dislocations. The relationship between grain size and corrosion resistance is more complicated than initially thought. Increased corrosion resistance can be achieved by finer grain size in low-carbon steel. The effective diffusion path for corrosive species, such as oxygen and chloride ions, to reach the metal surface is reduced due to smaller grain sizes. Furthermore, grain boundaries can be used as sites for localized corrosion, resulting in preferential attack in certain regions. The formation of corrosion-prone areas, such as grain boundaries and grain boundary precipitates, can result in decreased corrosion resistance if larger grain sizes are present. The material's degradation may be accelerated by the initiation and propagation of corrosion in these regions. The relationship between grain size and corrosion behavior in low-carbon steel has undergone extensive research. An optimal grain size has been discovered to increase corrosion resistance. The ideal grain size is usually between 10 and 100 < unk > m, depending on the specific corrosive environment. Also, it has been observed that various corrosion mechanisms can take place depending on the grain size. Uniform corrosion is often the dominant mechanism in low-carbon steels with fine grain sizes, for instance. In steels with larger grain sizes, localized corrosion phenomena, such as pitting corrosion or intergranular corrosion, may be more prevalent.

One of Pakistan's most well-organized industrial sectors is the sugar industry. In 2016–17, the sector produced close to 6–7 million tonnes of sugar^1^. Several compounds are used to extract sugar, which is mostly obtained from sugar cane and beet^2^.

The majority of industrial machinery is made of low-carbon (mild) steel^3^ and is typically advised for handling aqueous solutions in related industries such as sugar processing. Although the sugar is chemically rather inert, it becomes somewhat acidic during processing and boils at temperatures up to 100 °C^4^. Low-carbon steel is preferred for pipes in manufacturing industries because of its affordability and superior weldability, but it is very susceptible to acids and begins to degrade in an acidic environment (mainly juice). It has a low corrosion resistance, is easily machinable, has a good balance of strength and ductility, and contains between 0.01% and 0.25 percent carbon^5^. A material corrodes when it has a chemical interaction with its surroundings, which can include water, air, carbon dioxide, organic liquids, molten salts, or gaseous sulfur^6^. The rate of corrosion varies depending on the climate and the material's composition^7^.

Since corrosion-resistant materials have advanced, it still happens occasionally under specific circumstances. Low carbon steel's (mild steel) corrosion rate is affected by its microstructure in addition to electrolytic conditions^8^. The heterogeneities in the material cause small areas to have different potentials, which is what causes corrosion in aqueous media. These heterogeneities can be as small as an atom or as large as several hundred microns, and they can be caused by a number of things, including flaws in the crystal structure, chemically distinct phases, the segregation of elements or phases, and non-metallic inclusion^7,9^. Additionally, carbon steel of the same grade from various manufacturers can have dramatically varied compositional and microstructural characteristics, and this variation affects the corrosion rate^9,10^. The microstructure of low-carbon steel (AISI 1010) is crucial for understanding the rate and mechanism of corrosion.

The influence of electrical characteristics and chemical composition on corrosion behavior has been studied by numerous researchers^11,12^. Understanding the corrosion mechanism of low-carbon steel in a carbon dioxide atmosphere has received a lot of attention recently^13^. Morsy^14^ has also looked into how temperature and carbon dioxide partial pressure affect the rate of corrosion in relation to other factors^15^. However, there hasn’t been much research on how microstructure affects aqueous media. An empirical link between carbon steel corrosion and microstructure was found by Meshra et al.^16^.

In this work, low carbon steel (AISI 1010) with various microstructures formed during the heat treatment procedure had their impacts on corrosion resistance evaluated. By using potentiodynamic polarization studies to determine the electrochemical parameter for the material, surface morphology was examined using scanning electron microscopy (SEM), and analytical analysis was carried out using energy dispersive spectroscopy (EDS).

## Materials and methods

### Material

**Figure 1:**
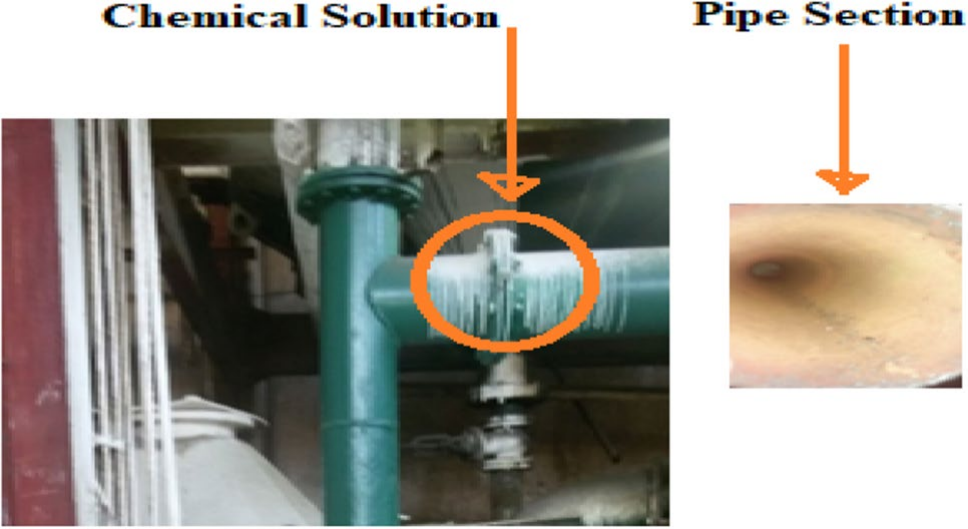
Corroded pipe section from sugar industry.

The material is commonly called low carbon steel AISI 1010 grade with chemical composition as follows: Fe (98.89%), C (0.09%), Mn (0.46%), P (0.08%), Si (0.39%), and S (0.09%). The material under investigation was collected from the Khazana Sugar Mill pipeline that was used to transport lime salt solution has collapsed because of internal corrosion while in service Fig. 1.

### Heat treatment

Heat treatment was carried out in a Muff furnace with a temperature of 880 °C–950 °C for 45 min and water was quenched, to produce different microstructures from the same material. The unsuccessful material was collected, divided into samples of 10 × 10 × 4 mm, and heated to harden it to change the grain sizes.

### Sample preparation

Seven heat-treated samples and seven “As Received (AR)” samples with a 10 mm × 10 mm dimension and a thickness of around 2 mm each were created. The samples were mounted in backlit powder at 150 kg/cm^2^ pressure, and 170 °C Temperature. By using (6, 3, and 0.25) µm diamond paste on polishing cloth, samples were finely polished and then etched in 2% Nital solution (2 ml HNO3 and 98 ml C2H5O) to reveal various structural properties of the material to get highly polished surfaces.

## Experimental results

### Microstructural analysis

#### SEM analysis

**Figure 2:**
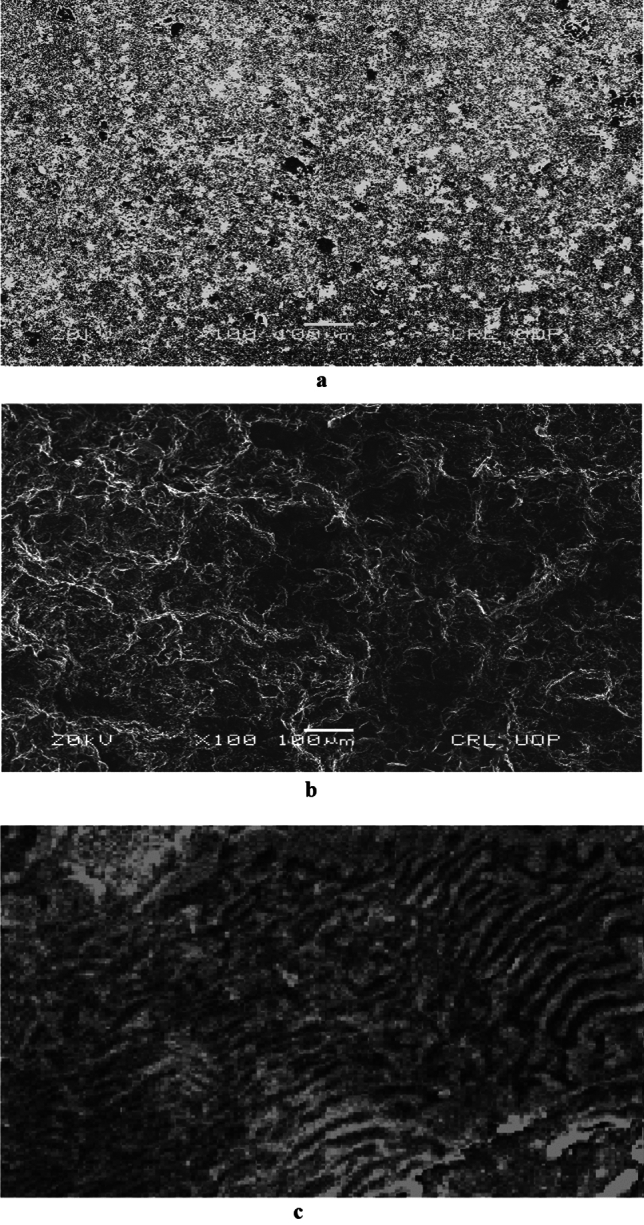
(**a**) SEM analysis of as received material. (**b**) SEM analysis of water quenched material. (**c**) SEM analysis of water quenched material.

The microstructure of both AR and heat-treated material was assessed using scanning electron microscopy SEM. The microstructure of AR material shown in Fig. 2a and b, consist of higher ferrite contents with a coarse grain structure. The ductility and softness of ferritic phases are higher when compared to pearlitic phases as shown in Fig. 2c.

**Figure 3:**
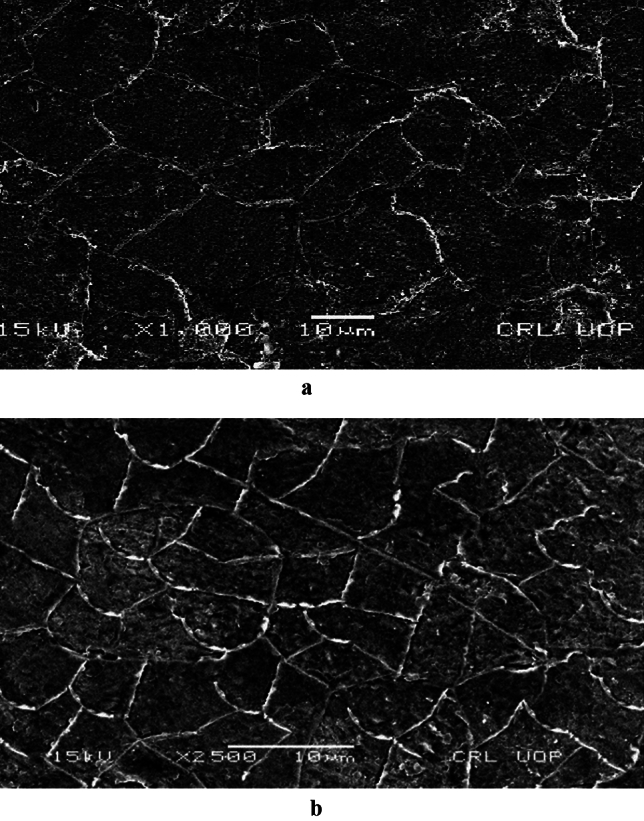
(**a**) Microstructure of as received material at 2000X magnification. (**b**) Microstructure of water quenched material.

The microstructure of as received AR and water-quenched WQ material is depicted in Fig. 3a and b respectively. It is seen from Fig. 3a that the grain size is bigger for the as-received material and gets finer with quenching. In Fig. 3b, where the martensitic structure is surrounded by a white ferrite network because the carbon didn't have enough time to become stuck in the solution and, disperse out of it.

### EDS analysis

**Figure 4:**
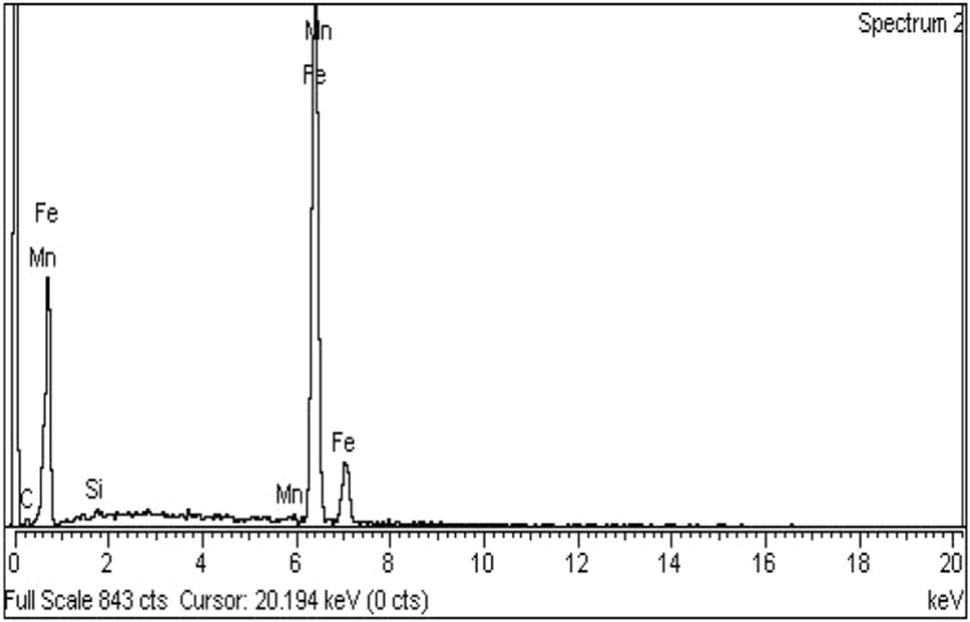
EDS analysis of low carbon (AISI 1010) steel.

To analyze the elemental composition of the substance, an EDS analysis was performed. When examining the attributes of low carbon steel (AISI 1010), the composition of other elements that are present in very small amounts was not taken into account because the properties of carbon steel are predominantly dependent on the carbon ingredient. Fe (98.89%), C (0.09%), Mn (0.46%), P (0.08%), Si (0.39%), and S (0.09%) make up the chemical breakdown of the components, which is depicted in Fig. 4.

### Grain size measurement

#### Planimetric (Jeffries)

The particle sizes were measured to determine the number of grains per square millimeter (mm2). Grain sizes are determined using the Jeffries Planimetric technique. Using the Jefferies formula, a circle with a diameter of 7.62 cm for 1000 magnification is sketched on the noticeable grains, and the number of grains (n1) interior of the circle and those "n2" that intersect the circle are counted to get the number of grains^17^;

$$No.\, of\, Grains = f \left( {n1 + n2 /2} \right)$$

where $$f = \frac{{M^{2} }}{Ac}$$

And

$$M = Magnification$$

$$Ac = Area\, of\, Circle$$

$$f = Jeffries\, Multiplier$$

**Figure 5:**
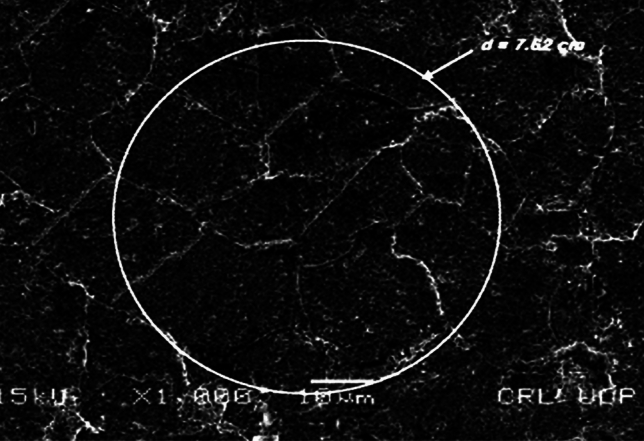
Average grain size of (as received) low carbon steel (AISI 1010) material.

**Figure 6:**
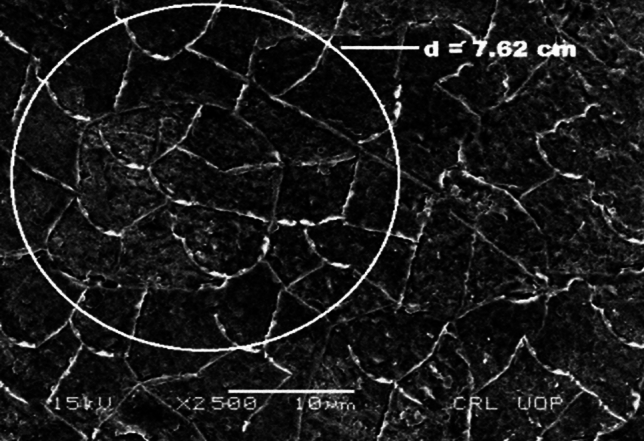
Grain sizes of water quenched low carbon steel (AISI 1010) material.

Finding the Jeffries multiplier ‘f’^17^ the average grain size 13 µm of AR material, as shown in Fig. 5. The WQ material is treated using the same process, as indicated in Fig. 6, and the average grain size was found to be 7 µm.

### Corrosion rate measurement

The constant immersion method and the potentiodynamic polarization method were used to determine the corrosion rate.

#### Constant immersion method

We used four samples of each of the water-quenched materials (WQ1, WQ2, WQ3, and WQ4) and four samples of the material as received (AR1, AR2, AR3, and AR4). Samples were prepared for the constant immersion method after the surface areas of the materials were measured using a Vernier caliper and the beginning weights were measured using an electronic weight balance with a measurement capacity of 0.001gm-5gm.

The complete specimen was submerged in moist soil, and the corrosion layers were carefully cleaned with kerosene oil every 15 days at intervals to prevent damage to the specimens. The weight loss of the samples was then calculated per unit area and plotted.

**Figure 7:**
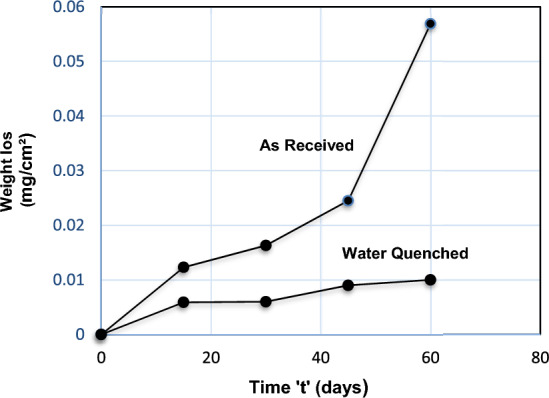
Comparison of weight loss by constant immersion of As-received and water quenched material.

It was determined that, as indicated in Fig. 7, the weight loss of (AR) material was significantly larger than that of (WQ) material.

#### Potentiodynamic polarization

An electrochemical test is performed in a cell with an electrolyte and three electrodes: a working electrode, a reference electrode or SCE, and a counter electrode, to which the potential is delivered. Testing samples were created by metallographic polishing and attaching copper wires with cold-setting glue. The electrolytic solution was only applied to a 1 cm2 area. When submerged in a corrosive medium, both oxidation and reduction processes take place. The specimen needs to have cathodic and anodic currents flowing across its surface. A net flow of charge occurs when the specimen is polarized. Plotting the data as potential vs. current is known as a Tafel plot. The software calculates the value of corrosion current based on a Tafel plot and then applies Faraday's Law to corrosion current to calculate the corrosion rate.

### Tafel plot

**Figure 8:**
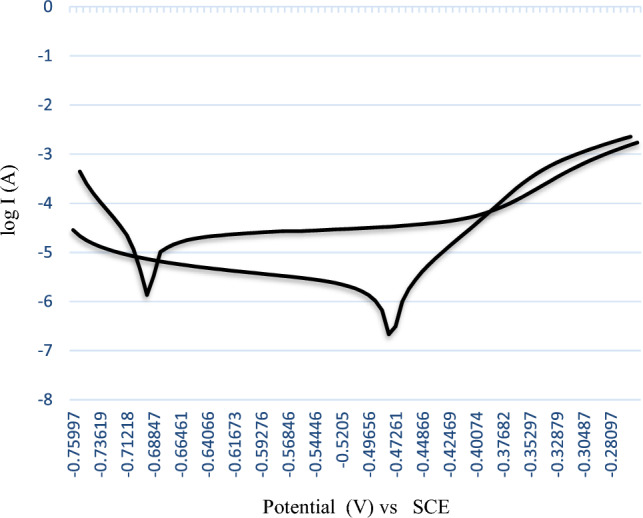
Comparison of polarization behavior of (As Received) and (Water quenched).

Icorr, the corrosion current, is measured using the Tafel plot. Tafel plots are created using the NOVA software, which is also used to determine corrosion current and the Tafel slope. The Tafel plots of (AR) and (WQ) materials are depicted in the following Fig. 8. Table 1 provides a summary of the numerical results, showing that the corrosion potential Ecorr of the (AR) material is − 702.0 mV, which is lower than the (WQ) material's Ecorr of − 493.0 mV. Also, it has been stated that the corrosion current density of (AR) material, Icorr, is 12.20 μA, significantly higher than that of (WQ) material, Icorr, which is 2.260 μA. Table 1 lists the values for the anodic Tafel constant βA and the cathodic Tafel constant βC.

**Table 1:** Data from Tafel Plot.

Sample	Ecorr (mV)	Icorr (µA)	C.R (mpy)
As received	− 702.0	12.20	5.580
Water quenched	− 493.0	2.260	1.032

The numerical data is summarized in Table 1 in which the corrosion potential Ecorr, of (AR) material is − 702.0 mV, lower than the corrosion potential Ecorr, of (WQ) material which is − 493.0 mV. It is also indicated that the corrosion current density of Icorr, of ‘AR’ material is 12.20 µA, much higher than Icorr, of (WQ) material which is 2.260 µA. Anodic Tafel constant βA, and cathodic Tafel constant βC, values are given in Table 1.

The corrosion rate was found to be 5.58mpy for the AR material and 1.032mpy for the WQ material according to Faraday’s Law.

## Discussion

By quenching, it is possible to control the grain size of low-carbon steel, which would result in a finer microstructure. Compared to the ferritic microstructure that was as-received, the finer microstructure had a lower corrosion rate. In addition, the study examined the impact of heat treatment on the corrosion rate of low-carbon steel. The as-recieved material was found to have a coarser microstructure and higher corrosion rate, compared to water quenched microstructure. coarser microstructure can make low-carbon steel more susceptible to corrosion, as indicated by this.

Grain size and structure play an important role in determining the corrosion behavior of low-carbon steel, as highlighted by the results of this study. The use of quenching can lead to a finer microstructure and lower corrosion rate, while annealing can result in a coarser microstructure and greater corrosion susceptibility. Industries and applications that depend on corrosion resistance for the performance and durability of low-carbon steel components can benefit from these findings.

This study examined the corrosion rates of various low-carbon steel microstructures. Grain size and structure had an impact on how low-carbon steel (AISI 1010) corroded. According to SEM investigation, the material's as-received microstructure was entirely ferritic Fig. 2a, and it is demonstrated in Fig. 3b that this microstructure increases finer with quenching.

When grain size was measured using the Jefferies Planimetric method, a significant difference was noticed. El-Sayed et al.^18^ has reported that quenching causes changes in grain structure from coarse to fine, which triggers recrystallization, and creates new grain at the expense of existing ones. Fine grain recrystallization is replaced with a material of coarser grains, of (AR). Microstructural research shows that quenching of ferritic materials, which have coarse-grain pearlitic and martensitic structures, results in a reduction in ferritic grain size and an increase in the number of grains. Two alternative approaches were used to study the impact of corrosion.

The corrosion phenomenon was carried out by constant immersion, of both the as-received and water quenched samples. The corrosion rate was determined by analyzing the decreases in weight per unit surface area by removing the corrosion product carefully from the surface each day and then finding out the average values, in which the entire specimens were evaluated, behaved particularly the different rates as indicated in Fig. 7. As the test duration lengthens, the rate of assault gradually reduces in tests of this type^13,13^, this is following the widely accepted facts that anytime a continuous and adhering corrosion product forms on a metal surface, it always creates a high or reduced implicit impediment against a consequent corrosion attack k^19^. The efficiency of the impediment depends on the regions that have high carbon contents (carbides), which serve as the anode, with the majority of the steel having smaller grain sizes, which serve as the cathode^15^. A Tafel curve^20^ of the specimen being studied is obtained after a potentiodynamic polarization test in a 3.5% NaCl aqueous solution at room temperature, as illustrated in Fig. 8. It was discovered that altering the microstructure morphology dramatically the corrosion rate. With a reduction in grain sizes, the polarization curve shows increased corrosion potential and decreased corrosion rates. This is due to the material's passivation, which will start readily when grain sizes are reduced. Passivation begins at the borders and eventually spreads to crystalline surface flaws. A larger density of grain boundaries and dislocations can be found inside smaller grains. The greater number of active sites needed to swiftly construct a continuous and protective passive layer will be made available by the increased fraction of grain boundaries. Ions or electrons have a harder time moving near the surface to take part in an electrochemical reaction because of the passive coating.

It is found that smaller grain size carbon steel has a larger passive layer percentage and low corrosion rate because there are more passive film nucleation sites. Vahid Afshari et al.^17^ also stated that more active atoms on the surface are assumed to be the cause of the positive shift in the circuit potential and polarization curve because they will participate in reactions and build a better passive layer that is protective of the surface. Figure 8, is the combination of the obtained data consisting comparison of polarization behavior of (As Received), and (Water quenched).

## Conclusion

In conclusion, grain size variations affect the corrosion rate of low-carbon steel (AISI 1010), which in turn affects the microstructural mechanical properties of the material. The effective diffusion path for corrosive species is reduced by smaller grain sizes, which generally improves corrosion resistance. Localized corrosion and decreased corrosion resistance may be a result of larger grain sizes. To maximize the corrosion resistance of low-carbon steels, it is crucial to find the optimal grain size.

This research has compellingly illustrated the profound influence of microstructural variations on corrosion and penetration rates. The comparative analysis between fine-grain carbon steel and coarse-grain steel unequivocally underscores the susceptibility of the latter to corrosion. The study has brought to the forefront the pivotal role played by two distinct heat treatments in shaping corrosion behavior and the overall efficacy of corrosion resistance in low-carbon (AISI 1010) steel. Furthermore, it underscores the paramount importance of employing complementary surface analysis techniques, such as Scanning Electron Microscopy (SEM) and Energy-Dispersive X-ray Spectroscopy (EDS), to meticulously delineate surface morphology and better inform corrosion mitigation strategies.

## Data Availability

The datasets used and/or analyzed during the current study are available from the corresponding author upon reasonable request.
